# Perceptions and psychosocial correlates of e-cigarette use among Hong Kong university students: A mixed-methods study

**DOI:** 10.18332/tid/226575

**Published:** 2026-10-22

**Authors:** Zecheng Mai

**Affiliations:** 1 Department of Sociology, Hong Kong Shue Yan University, Hong Kong SAR, China

**Keywords:** university students, psychosocial factors, e-cigarettes, Hong Kong, peer influence

## Abstract

**INTRODUCTION:**

E-cigarette use among young adults is a concern in Hong Kong, where alternative smoking products have been prohibited since 2022, with public possession banned from 2026. Evidence among undergraduates is limited. This study examined e-cigarette use and associated psychosocial factors among Hong Kong undergraduates.

**METHODS:**

A sequential explanatory mixed-methods study was conducted at a Hong Kong university. Phase 1 was a cross-sectional survey (n=267) of full-time undergraduates aged ≥18 years, using convenience sampling. Measures, guided by an extended Theory of Planned Behavior framework, included peer influence, acceptability of public use, and perceived surveillance (1–5 scales). Outcomes were ever e-cigarette use during ages 13–19 years and current (past-30-day) use, analyzed by multivariable logistic regression (AOR, 95% CI). Phase 2 comprised 21 interviews, analyzed inductively.

**RESULTS:**

Mean age was 20.8 years (SD=2.0); 55.4% were female. Ever use during ages 13–19 years was 12.4% (33/266) and current use 8.6% (23/266); 86.8% of ever e-cigarette users had also smoked. Peer influence was associated with ever use (AOR=3.34; 95% CI: 1.95–5.73; p<0.001) and current use (AOR=3.20; 95% CI: 1.86–5.49; p<0.001); female sex was inversely associated with ever use (AOR=0.25; 95% CI: 0.09–0.65; p=0.005). Current use was also associated with acceptability of public use (AOR=1.97; 95% CI: 1.08–3.61; p=0.028) and inversely with surveillance (AOR=0.63; 95% CI: 0.41–0.97; p=0.034). Stress relief and peer influence were the most reported reasons for use (43.4% each), and friends the most common source (67.9%). Regulatory deterrence did not differ between users and non-users (p=0.683) despite high policy awareness (89.9%). Interviews described initiation through friends, use under stress, and acquisition through friends and cross-border sourcing.

**CONCLUSIONS:**

Peer influence was most strongly associated with e-cigarette use; perceived legal deterrence was not. Because this cross-sectional design cannot establish temporal order, further studies are needed to determine causality and characterize informal supply channels among university students.

## INTRODUCTION

Electronic cigarettes (e-cigarettes) have become a growing public health concern among adolescents and young adults worldwide, and global health authorities have identified youth vaping as a priority for tobacco control^1^. Prevalence has risen across many jurisdictions: past-30-day use among United States high school students increased from 1.5% in 2011 to 20.8% in 2018^2^, and increases have also been documented across 17 European study sites in the Global Youth Tobacco Survey^3^. Concern about these trends reflects evidence of respiratory and other toxic effects of e-cigarette aerosol in adolescents and young adults^4^, and evidence from systematic reviews and meta-analyses that e-cigarette use among non-smoking young people is associated with subsequent initiation of combustible cigarette smoking^5,6^.

In Hong Kong, these concerns arise within one of the region’s most restrictive tobacco-control environments. Since 30 April 2022, the import, promotion, manufacture, sale and commercial possession of alternative smoking products (ASPs), including e-cigarettes, have been prohibited^7^, and from 30 April 2026 possession of specified ASPs in a public place also became an offense, with penalties ranging from a fixed penalty for small quantities to a maximum fine and imprisonment for larger quantities^8^.

Despite this legal framework, e-cigarette experimentation has not disappeared among young people in Hong Kong. A territory-wide survey of secondary school students found that current use was associated with male sex, weaker knowledge of smoking harms, cigarette smoking, use of other tobacco products and alcohol consumption^9^. Among young adults aged 18–35 years in Hong Kong, e-cigarettes were commonly perceived as less harmful and less addictive than conventional cigarettes, and these perceptions were associated with use and with susceptibility to future use^10^. School-level prevalence of e-cigarette use has likewise been associated with individual-level susceptibility and tobacco use behaviors in Hong Kong adolescents^11^, and exposure to e-cigarette advertising has been associated with more favorable attitudes and greater susceptibility among never users^12^. Together, these findings indicate that psychosocial influences may remain relevant even where formal market access is heavily restricted.

The Theory of Planned Behavior (TPB) offers a framework for understanding such behavior, proposing that attitudes, subjective norms and perceived behavioral control shape intention and subsequent action^13^. Systematic reviews of TPB applications to e-cigarette use among adolescents report that favorable attitudes and social norms are the constructs most consistently associated with intention and use, while findings for perceived behavioral control are mixed^14^, and meta-analytic work supports the model’s applicability to smoking behavior more broadly^15,16^. Applied to e-cigarettes, favorable perceptions of vaping and peer influence may each be associated with experimentation and continued use^17,18^. Socio-ecological reviews similarly identify peer norms and psychological distress as recurrent factors associated with use among adolescents and young adults^19^, and both perceived stress and stress-related reasons for vaping have been associated with e-cigarette use in emerging adults^20,21^. Qualitative work indicates that young people may recognize addiction risk while continuing to use products they find appealing and easy to conceal^22^. Perceptions of relative harm and of chemical content are also associated with interest in, and use of, e-cigarettes^23^, and device and product characteristics – including high-nicotine pod devices and sweet flavors – have been linked to appeal among young people^24,25^, as has exposure to e-cigarette marketing on social media^26^.

In a prohibitionist setting, the policy environment may add two further dimensions that are not part of the standard TPB. Drawing on deterrence theory^27^, perceived regulatory deterrence refers to the expected certainty and severity of punishment for a violation, and perceived surveillance refers to the sense of being monitored or at risk of detection in public or near-campus settings. Comparative evidence indicates that the regulatory environment is associated with how e-cigarettes are used and with the outcomes of that use^28^, and commentators have argued that policy design must weigh youth protection against other public health considerations^29^. Historical analysis of youth substance use in Hong Kong further suggests that restrictive policy has tended to shift the visibility and geography of use rather than eliminate it^30^.

Most Hong Kong studies to date have focused on secondary school students^9,11,12^ or on broader young adult populations^10^, rather than on university undergraduates specifically, and research integrating TPB-related psychosocial factors with regulatory perceptions in the post-ban Hong Kong context is limited. The present study therefore examined the prevalence, perceptions, and psychosocial factors associated with e-cigarette use among undergraduates at one university in Hong Kong using a sequential explanatory mixed-methods design.

## METHODS

### Study design, setting and period

This study used a sequential explanatory mixed-methods design with two connected phases. Phase 1 was a cross-sectional online survey collecting primarily quantitative data; Phase 2 comprised semi-structured follow-up interviews with a purposive subsample of survey participants, conducted to explain and deepen the quantitative findings. The study was conducted at one self-financing university in the Hong Kong Special Administrative Region, China. Survey data were collected between January and March 2026, and interviews between January and February 2026.

### Participants and eligibility criteria

The target population comprised full-time undergraduate students at the participating university. Inclusion criteria were: 1) current enrolment as a full-time undergraduate; 2) age ≥18 years; 3) ability to provide informed consent; and 4) ability to complete the questionnaire in either English or Traditional Chinese. Students who were not undergraduates, and those aged <18 years, were excluded.

### Sampling and recruitment

The survey used convenience sampling with university-wide recruitment and light quota monitoring intended to approximate the undergraduate population profile by gender, year of study, and local versus non-local secondary education background. The target sample size was 250–300 valid responses; 267 valid responses were obtained. Recruitment was conducted exclusively through official university channels: university-wide emails were sent to students’ official campus email addresses following approval by the relevant institutional bodies. No public social media advertisements were used. Participants accessed an information sheet and an electronic consent page before entering the survey. Of the 267 respondents, 56 (21.0%) indicated willingness to be interviewed and 49 provided contact details through a separate online form stored independently of the survey dataset; 21 interviews were subsequently conducted.

For Phase 2, purposive sampling was used to obtain variation across current e-cigarette users, ever-but-not-current users and never users, and across local and non-local secondary educational backgrounds. Interviews lasted 15–30 minutes.

### Measures

The questionnaire was developed in English and translated into Traditional Chinese using forward–backward translation with reconciliation by two independent bilingual reviewers. A pilot test with 10–15 undergraduates assessed item comprehension, completion time and wording sensitivity; minor revisions followed. All policy items referred to the restrictions in force since 30 April 2022 and explicitly included the public-place possession prohibition effective 30 April 2026.

*Outcome variables*

Two binary outcomes were analyzed.

Ever e-cigarette use during ages 13–19 years was defined as any self-reported use of an e-cigarette between the ages of 13 and 19 years (0=no; 1=yes); one respondent answering ‘cannot remember’ was treated as missing.

Current e-cigarette use was defined as use on at least one of the 30 days preceding the survey (0=no; 1=yes), derived from a free-text count of days.

A secondary outcome, lifetime ever e-cigarette use, was defined as reporting use during ages 13–19 years, or any use in the past 30 days, or a valid age at first e-cigarette use.

*Sociodemographic variables*

Gender (male, female, prefer not to say); age in completed years (continuous); year of study (Year 1, Year 2, Year 3, Year 4); location of secondary schooling (Hong Kong SAR , Mainland China, overseas); and current living arrangement (family home, student hostel or shared flat, alone or other).

*Tobacco use history and household exposure*

Ever conventional cigarette smoking (yes, no); past-30-day conventional cigarette smoking (yes, no); dual ever use, defined as ever use of both products; any family member who regularly smokes conventional cigarettes (yes, no); and any family member or partner who uses e-cigarettes (yes, no).

*Psychosocial variables*

Items were scored on 5-point Likert scales (1=strongly disagree to 5=strongly agree; for the acceptability items, 1=totally unacceptable to 5=totally acceptable). Composite scores were calculated as the mean of the constituent items, giving a possible range of 1 to 5, with higher scores indicating a higher level of the construct.

Peer influence (subjective norms; 3 items): ‘Many of my friends use e-cigarettes’; ‘The more friends around me who use e-cigarettes, the more I want to try or continue using them’; ‘If my friends invite me to vape, it is hard for me to say no’. Possible range 1–5; Cronbach’s α=0.78.

Acceptability of e-cigarette use in public settings (attitudes; 4 items): acceptability of use on the street or in parks, in restaurants or cafés, in outdoor areas on campus, and in indoor public places. Possible range 1–5; Cronbach’s α=0.81.

Perceived surveillance (two items): ‘If I use e-cigarettes in a no-smoking area, I think there is a high chance of being noticed or reported’; ‘I feel that the university or government closely monitors the use of e-cigarettes in public places’; α=0.77 among those answering both. Because the questionnaire instructed respondents for whom the block was ‘not applicable’ to skip it, the first item was answered by only 78 respondents, whereas the second was answered by all 267. The second item alone was therefore used as the measure of perceived surveillance in all analyses of the full sample, and the two-item composite is reported descriptively for the subgroup who answered both.

Perceived regulatory deterrence (2 items): ‘Because I am worried about being fined or prosecuted, I have reduced or stopped using e-cigarettes’; ‘The government’s tougher stance on e-cigarettes makes me less willing to use them in public’; possible range 1–5; α=0.88). These items were answered by 75 respondents (28.1%).

Perceived harm, stress and stigma items: Perceived harm was measured by two items ‘I worry about my health if I use e-cigarettes for a long time’; ‘I feel that e-cigarettes have little impact on my physical and mental health’. These items were not internally consistent (inter-item r= -0.02) and are therefore reported separately rather than as a composite. Perceived stress in the past month and use of e-cigarettes to cope with negative emotions were likewise reported as separate items (inter-item r=0.32). Three stigma and concealment items were also collected (α=0.81).

Policy awareness: was a single item with three response options – aware, has heard of it but unsure of the details , not aware.

*Handling of conditional non-response*

The perceived-harm, stress, stigma and deterrence blocks each carried the instruction ‘those who are not applicable, please skip’. Response to these blocks was therefore strongly related to e-cigarette use status: the deterrence items, for example, were answered by 46 of 53 (86.8%) respondents who had ever used an e-cigarette but by only 29 of 214 (13.6%) who had not. Because non-response to these items is not independent of the outcome, these variables were not entered into the regression models; they are reported descriptively. Only variables answered by the whole sample – peer influence, acceptability of use in public settings, the second surveillance item, sociodemographic characteristics and smoking history – were used as explanatory variables.

*Potential confounding factors*

Age and gender were specified *a priori* as potential confounding factors and were included in the primary models. Year of study, location of secondary schooling, living arrangement, family smoking and family or partner e-cigarette use, were examined in sensitivity models.

### Data collection

Survey data were collected through a mobile-optimized online platform. No names, student identification numbers, or other directly identifying information were collected in the survey. Interviews were audio-recorded only after separate consent was obtained, and recordings were transcribed verbatim and de-identified before analysis.

### Statistical analysis

Analyses were conducted in SPSS version 29.0 (IBM Corp., Armonk, NY, USA) – amend to match the software actually used for the final analysis. Free-text responses to the two ‘number of days in the past 30 days’ items were coded numerically; explicit statements of no use were coded as zero and ranges were coded at their midpoint. Three age entries that were non-numeric or biologically implausible were treated as missing, leaving 264 valid age values. Categorical variables are summarized as frequencies and percentages, and continuous variables as means with standard deviations (SDs). Prevalence estimates are presented with Wilson 95% confidence intervals (CIs). Internal consistency of multi-item measures was assessed with Cronbach’s α.

Bivariate associations were examined using Pearson chi-squared tests for categorical variables and Welch’s independent-samples t-tests for continuous variables. Two binary logistic regression models were then fitted, with ever e-cigarette use during ages 13–19 years as the outcome in Model 1 and current (past-30-day) use in Model 2. Both models contained the same five variables, entered simultaneously: peer influence, acceptability of use in public settings and perceived surveillance (each modeled as a continuous score, with odds ratios expressed per 1-point increase), age (per year) and gender. The number of variables was limited by the number of outcome events in order to avoid overfitting. Ten respondents who preferred not to state their gender were excluded from the regression models because no outcome events occurred in that group; the analytic samples were therefore 254 and 255 respondents. Crude odds ratios (OR) were obtained from univariable models fitted in the same analytic sample; adjusted odds ratios (AORs) were obtained from the full multivariable models. Both are presented with 95% CIs and p-values, including for variables that did not reach statistical significance. Model fit was assessed using the Nagelkerke R^2^, the likelihood-ratio test and the Hosmer–Lemeshow goodness-of-fit test. Sensitivity analyses: 1) retained all 267 respondents using Firth’s penalized maximum likelihood to accommodate the zero-event gender category; 2) added year of study, location of secondary schooling, living arrangement, family smoking and family or partner e-cigarette use; and 3) additionally adjusted for ever conventional cigarette smoking. Statistical significance was set at p<0.05, two-tailed.

### Qualitative analysis

Open-ended survey responses and interview transcripts were analyzed inductively using NVivo 14. Fifty-two respondents (19.5%) provided at least one substantive open-ended response. Initial codes were generated from the data and then grouped into broader themes. Peer debriefing with the research supervisor was used to strengthen trustworthiness; member checking was not feasible within the study timeline. Findings from the two components were integrated at the interpretation stage.

### Ethics

The study was approved by the Department Research Ethics Committee of the Hong Kong Shue Yan University on 27 November 2025. Participation was voluntary throughout; all participants provided informed consent, and participants could withdraw at any time without consequence.

## RESULTS

### Sample characteristics

A total of 267 valid survey responses were analyzed (Table 1). The mean age of participants was 20.8 years (SD=2.0; n=264; range 18–30); 55.4% (148/267) were female, 40.8% (109/267) were male, and 3.7% (10/267) preferred not to state their gender. By year of study, 24.0% (64/267) were in Year 1, 20.2% (54/267) in Year 2, 36.3% (97/267) in Year 3 and 19.5% (52/267) in Year 4. Most had attended secondary school in Hong Kong SAR (67.4%; 180/267), with 32.2% (86/267) in Mainland China. Two-thirds lived in the family home (65.9%; 176/267) and 32.2% (86/267) in a student hostel or shared flat.

**Table 1:** Sociodemographic characteristics of undergraduate survey participants, a cross-sectional survey at one university in Hong Kong Special Administrative Region, China, 2026 (N=267)

*Variables*	*n*	*%*
**Gender**		
Male	109	40.8
Female	148	55.4
Prefer not to say	10	3.7
**Age** (years) (N=264)^a^ mean (SD) [range]	20.8 (2.0 ) [18–30]	
**Year of study**		
1	64	24.0
2	54	20.2
3	97	36.3
4	52	19.5
**Location of secondary schooling**		
Hong Kong SAR	180	67.4
Mainland China	86	32.2
Overseas	1	0.4
**Current living arrangement**		
Family home	176	65.9
Student hostel or shared flat	86	32.2
Alone or other	5	1.9
**Family member smokes conventional cigarettes**		
No	155	58.1
Yes	112	41.9
**Family member or partner uses e-cigarettes**		
No	231	86.5
Yes	36	13.5

SD: standard deviation. SAR: Special Administrative Region. Percentages may not sum to 100% because of rounding.

a Three age entries were non-numeric or biologically implausible and were treated as missing.

### Prevalence and patterns of use

Prevalence estimates are shown in Table 2. Ever e-cigarette use during ages 13–19 years was reported by 12.4% of respondents (33/266; 95% CI: 9.0–16.9) and current (past-30-day) use by 8.6% (23/266; 95% CI: 5.8–12.6). Lifetime ever e-cigarette use was 19.9% (53/267; 95% CI: 15.5–25.0). Ever conventional cigarette use was also 19.9% (53/267), past-30-day conventional cigarette use 13.9% (37/266; 95% CI: 10.3–18.6), and dual ever use of both products 17.2% (46/267; 95% CI: 13.2–22.2). Of the 53 respondents reporting lifetime ever e-cigarette use, 46 (86.8%) had also ever smoked conventional cigarettes; of the 214 respondents who had never used an e-cigarette, 7 (3.3%) had ever smoked conventional cigarettes.

**Table 2:** Prevalence of e-cigarette and conventional cigarette use, patterns of use, psychosocial measures and policy awareness, a cross-sectional survey of undergraduates at one university in Hong Kong Special Administrative Region, China, 2026 (N=267)

*Variables*	*n/N*	*% (95% CI)*
**Panel A. Product use prevalence**		
Ever e-cigarette use (age 13–19 years)	33/266	12.4 (9.0–16.9)
Lifetime ever e-cigarette use	53/267	19.9 (15.5–25.0)
Current (past-30-day) e-cigarette use	23/266	8.6 (5.8–12.6)
Ever conventional cigarette use	53/267	19.9 (15.5–25.0)
Past-30-day conventional cigarette use	37/266	13.9 (10.3–18.6)
Dual ever use (both products)	46/267	17.2 (13.2–22.2)
**Panel B. Reasons for use** (lifetime ever users, N=53)^a^		%
Stress relief or relaxation	23/53	43.4
Friends use them (peer influence)	23/53	43.4
Like the taste or flavor variety	21/53	39.6
Curiosity	19/53	35.8
Fun, fashion or trend	9/53	17.0
To help quit conventional cigarettes	8/53	15.1
Believed less harmful than cigarettes	7/53	13.2
Cheaper or easier to access	6/53	11.3
**Panel C. Acquisition channels** (lifetime ever users, N=53)^a^		
Friends gave or purchased on respondent’s behalf	36/53	67.9
Online platforms	14/53	26.4
Physical shops (before the ban)	10/53	18.9
Parallel imports or black-market channels	4/53	7.5
**Panel D. Settings of use** (lifetime ever users, N=53)^a^		
Bars/restaurants	25/53	47.2
At home	21/53	39.6
Public places (streets, parks)	20/53	37.7
At a friend’s home	19/53	35.8
Near or around campus	18/53	34.0
**Panel E. Psychosocial measures** (possible range 1–5)		**Mean (SD)**
Peer influence (3 items, α=0.78)	267	1.71 (0.90)
Acceptability of use in public settings (4 items, α=0.81)	267	1.78 (0.85)
Perceived surveillance, single item answered by all	267	3.63 (1.36)
Perceived surveillance, 2-item composite (α=0.77)^b^	78	3.66 (1.33)
Perceived regulatory deterrence (2 items, α=0.88)^b^	75	3.23 (1.55)
Stigma and concealment (3 items, α=0.81)^b^	152	3.21 (1.21)
Worry about long-term health effects (single item)^b^	131	4.15 (1.34)
E-cigarettes have little impact on health (single item)^b^	129	1.85 (1.25)
Perceived stress in past month (single item)^b^	150	3.05 (1.35)
Use e-cigarettes to cope with negative emotions (single item)^b^	132	1.67 (1.27)
**Panel F. Policy awareness**		%
Aware of the restrictions	155/267	58.1
Has heard of them but unsure of the details	85/267	31.8
Not aware	27/267	10.1

SD: standard deviation. CI: confidence interval. α: Cronbach’s alpha. Confidence intervals for prevalence are Wilson intervals.

a Percentages in Panels B–D are based on lifetime ever users and may exceed 100% because multiple responses were permitted.

b These items carried the instruction ‘those who are not applicable, please skip’ and were therefore answered by a subset of respondents; response was strongly related to e-cigarette use status (see Methods), and these measures were not used as explanatory variables in the regression models.

Among the 33 respondents reporting use during ages 13–19 years, frequency was most often low: 21 reported use ‘only once or a few times’ and 8 ‘less than once a month’, while 6 reported use 1–6 times per week and 5 almost every day.

Among the 53 respondents reporting lifetime ever use (multiple responses permitted), the most frequently reported reasons for use were stress relief (43.4%; 23/53), peer influence (43.4%; 23/53), flavor or taste (39.6%; 21/53) and curiosity (35.8%; 19/53). The most frequently reported acquisition channels were friends who gave or purchased e-cigarettes on the respondent’s behalf (67.9%; 36/53), online platforms (26.4%; 14/53), physical shops before the ban (18.9%; 10/53) and parallel-import or black-market channels (7.5%; 4/53). The settings most often reported were bars or restaurants (47.2%; 25/53), the respondent’s home (39.6%; 21/53), public places such as streets and parks (37.7%; 20/53), a friend’s home (35.8%; 19/53) and near or around campus (34.0%; 18/53) (Table 2).

Regarding policy awareness, 58.1% (155/267) reported being aware that Hong Kong has banned the import, sale and promotion of e-cigarettes, 31.8% (85/267) had heard of the restrictions but were unsure of the details, and 10.1% (27/267) were not aware; 89.9% (240/267) were therefore aware of the restrictions or had at least heard of them.

### Psychosocial measures and bivariate associations

Composite and item scores are shown in Table 2. Across the whole sample, the mean peer influence score was 1.71 (SD=0.90), and the mean acceptability score was 1.78 (SD=0.85), both on scales with a possible range of 1–5. The mean score on the perceived-surveillance item answered by all respondents was 3.63 (SD=1.36). Among the 75 respondents who answered the perceived regulatory deterrence items, the mean was 3.23 (SD=1.55), and among the 78 who answered both surveillance items, the two-item mean was 3.66 (SD=1.33).

In bivariate analyses, respondents reporting ever use during ages 13–19 years had higher mean peer influence scores than those who did not (2.95 vs 1.53; t=6.81, p<0.001) and higher acceptability scores (2.57 vs 1.67; t=4.78, p<0.001). Ever use during ages 13–19 years was more frequent among male than female respondents (21.1%, 23/109 vs 6.8%, 10/147; χ^2^=13.25, df=2, p=0.001) and among those who had ever smoked conventional cigarettes (58.5%, 31/53 vs 0.9%, 2/213; χ^2^=124.11, df=1, p<0.001). It did not differ significantly by year of study (χ^2^=3.35, df=3, p=0.341), location of secondary schooling (χ^2^=0.97, df=2, p=0.615) or living arrangement (χ^2^=1.44, df=2, p=0.486). Current use showed the same pattern for peer influence (3.12 vs 1.58; t=6.70, p<0.001) and acceptability (2.86 vs 1.68; t=5.09, p<0.001) but did not differ by gender (χ^2^=0.89, df=2, p=0.640).

Among respondents who answered the conditional item blocks, those who had ever used an e-cigarette scored higher than those who had not on the belief that e-cigarettes have little impact on health (2.74 vs 1.29; t=6.98, p<0.001), on using e-cigarettes to relax (3.22 vs 1.25; t=9.35, p<0.001) and on using e-cigarettes to cope with negative emotions (2.59 vs 1.13; t=6.21, p<0.001), whereas the two groups did not differ on worry about long-term health effects (4.20 vs 4.12; t=0.34, p=0.733) or on general perceived stress in the past month (2.94 vs 3.10; t= -0.66, p=0.511). Perceived regulatory deterrence did not differ between the two groups (3.29 vs 3.14; t=0.41, p=0.683). These comparisons are restricted to respondents who answered the relevant items and are subject to differential non-response.

### Factors associated with ever e-cigarette use during ages 13–19 years

Table 3 presents crude and adjusted odds ratios for ever e-cigarette use during ages 13–19 (n=254; 32 events). In the adjusted model, each 1-point increase in the peer influence score was associated with higher odds of ever use (AOR=3.34; 95% CI: 1.95–5.73; p<0.001), and female sex was associated with lower odds than male sex (AOR=0.25; 95% CI: 0.09–0.65; p=0.005). Acceptability of use in public settings (AOR=1.31; 95% CI: 0.73–2.33; p=0.362), perceived surveillance (AOR=0.77; 95% CI: 0.55–1.08; p=0.129) and age (AOR=1.02; 95% CI: 0.82–1.27; p=0.855) were not associated with this outcome after adjustment. The model was statistically significant (likelihood-ratio χ^2^=62.77, df=5, p<0.001), explained a moderate proportion of variance (Nagelkerke R^2^=0.412) and showed adequate calibration (Hosmer–Lemeshow χ^2^=8.03, df=8, p=0.431).

**Table 3:** Crude and adjusted odds ratios for ever e-cigarette use during ages 13–19 years, binary logistic regression, a cross-sectional survey of undergraduates at one university in Hong Kong Special Administrative Region, China, 2026 (N=254; 32 events)

*Variables*	*Ever users* *(N=32)* *Mean (SD)*	*Non-users* *(N=222)* *Mean (SD)*	*OR (95% CI)*	*p*	*AOR (95% CI)*	*p*
Peer influence	2.92 (1.17)	1.55 (0.71)	3.87 (2.52–5.93)	<0.001	3.34 (1.95–5.73)	<0.001
Acceptability of use in public settings	2.55 (1.05)	1.68 (0.77)	2.62 (1.75–3.91)	<0.001	1.31 (0.73–2.33)	0.362
Perceived surveillance	3.16 (1.42)	3.70 (1.36)	0.76 (0.59–0.99)	0.039	0.77 (0.55–1.08)	0.129
Age (years)	21.38 (1.88)	20.75 (2.01)	1.15 (0.97–1.37)	0.103	1.02 (0.82–1.27)	0.855
**Gender**, n (%)						
Male (ref.)	23 (21.1)	86 (78.9)	1.00		1.00	
Female	9 (6.2)	136 (93.8)	0.25 (0.11–0.56)	0.001	0.25 (0.09–0.65)	0.005

AOR: adjusted odds ratio. CI: confidence interval. SD: standard deviation. ref.: reference category. SAR: Special Administrative Region. Outcome: self-reported ever use of an e-cigarette between the ages of 13 and 19 years. Peer influence, acceptability and perceived surveillance were measured on scales with a possible range of 1–5; odds ratios are expressed per 1-point increase. Percentages for gender are row percentages. Crude ORs were obtained from univariable logistic regression models fitted in the same analytic sample. AORs were obtained from a single multivariable logistic regression model containing all five variables listed, entered simultaneously. Ten respondents who preferred not to state their gender were excluded because no outcome events occurred in that group; a sensitivity analysis retaining all respondents using Firth’s penalized maximum likelihood gave a peer influence AOR of 3.09 (95% CI: 1.85–5.18). Nagelkerke R²=0.412; likelihood-ratio χ^2^=62.77, df=5, p<0.001; Hosmer–Lemeshow χ^2^=8.03, df=8, p=0.431.

### Factors associated with current e-cigarette use

Table 4 presents crude and adjusted odds ratios for current (past-30-day) e-cigarette use (n=255; 23 events). In the adjusted model, peer influence was associated with current use (AOR=3.20; 95% CI: 1.86–5.49; p<0.001), as was acceptability of use in public settings (AOR=1.97; 95% CI: 1.08–3.61; p=0.028). Perceived surveillance was inversely associated with current use (AOR=0.63; 95% CI: 0.41–0.97; p=0.034). Age (AOR=0.96; 95% CI: 0.73–1.26; p=0.759) and female sex (AOR=1.86; 95% CI: 0.60–5.75; p=0.284) were not associated with current use. The model was statistically significant (likelihood-ratio χ^2^=56.72, df=5, p<0.001; Nagelkerke R^2^=0.439) with adequate calibration (Hosmer–Lemeshow χ^2^=7.60, df=8, p=0.473).

**Table 4:** Crude and adjusted odds ratios for current (past-30-day) e-cigarette use, binary logistic regression, a cross-sectional survey of undergraduates at one university in Hong Kong Special Administrative Region, China, 2026 (N=255; 23 events)

*Variables*	*Current users* *(N=23)* *Mean (SD)*	*Non-users* *(N=232)* *Mean (SD)*	*OR (95% CI)*	*p*	*AOR (95% CI)*	*p*
Peer influence	3.12 (1.08)	1.58 (0.76)	4.04 (2.53–6.47)	<0.001	3.20 (1.86–5.49)	<0.001
Acceptability of use in public settings	2.86 (1.08)	1.69 (0.75)	3.40 (2.12–5.44)	<0.001	1.97 (1.08–3.61)	0.028
Perceived surveillance	2.87 (1.32)	3.70 (1.36)	0.66 (0.49–0.89)	0.007	0.63 (0.41–0.97)	0.034
Age (years)	21.22 (1.48)	20.79 (2.04)	1.10 (0.90–1.35)	0.331	0.96 (0.73–1.26)	0.759
**Gender**, n (%)						
Male (ref.)	10 (9.2)	99 (90.8)	1.00		1.00	
Female	13 (8.9)	133 (91.1)	0.97 (0.41–2.30)	0.941	1.86 (0.60–5.75)	0.284

AOR: adjusted odds ratio. CI: confidence interval. SD: standard deviation. ref.: reference category. SAR: Special Administrative Region. Outcome: self-reported e-cigarette use on at least one of the 30 days preceding the survey. Peer influence, acceptability and perceived surveillance were measured on scales with a possible range of 1–5; odds ratios are expressed per 1-point increase. Percentages for gender are row percentages. Crude ORs were obtained from univariable logistic regression models fitted in the same analytic sample. AORs were obtained from a single multivariable logistic regression model containing all five variables listed, entered simultaneously. Ten respondents who preferred not to state their gender were excluded because no outcome events occurred in that group; a sensitivity analysis retaining all respondents using Firth’s penalized maximum likelihood gave a peer influence AOR of 2.94 (95% CI: 1.76–4.93) and a perceived surveillance AOR of 0.65 (95% CI: 0.44–0.96). Nagelkerke R^2^=0.439; likelihood-ratio χ^2^=56.72, df=5, p<0.001; Hosmer–Lemeshow χ^2^=7.60, df=8, p=0.473.

### Sensitivity analyses

Three sets of sensitivity analyses were conducted. First, retaining all 267 respondents and using Firth’s penalized maximum likelihood produced closely similar estimates (ever use during ages 13–19 years: peer influence AOR=3.09; 95% CI: 1.85–5.18; current use: peer influence AOR=2.94; 95% CI: 1.76–4.93; perceived surveillance AOR=0.65; 95% CI: 0.44–0.96). Second, adding year of study, location of secondary schooling, living arrangement, family smoking and family or partner e-cigarette use did not materially change the estimate for peer influence (ever use AOR=3.20; 95% CI: 1.80–5.69; current use AOR=2.54; 95% CI: 1.43–4.50), although the number of variables exceeded what the event count supports. Third, additional adjustment for ever conventional cigarette smoking attenuated the association with peer influence towards the null (ever use AOR=1.00; 95% CI: 0.53–1.90; current use AOR=1.39; 95% CI: 0.74–2.63), while ever conventional cigarette smoking itself showed a very large association with both outcomes (ever use during ages 13–19 years: AOR=83.45; 95% CI: 16.85–413.29). Because only 2 of 213 respondents who had never smoked conventional cigarettes reported e-cigarette use during the ages of 13–19 years, this model is close to complete separation and is effectively estimated within cigarette smokers; it is reported for transparency rather than as a preferred specification.

### Qualitative findings

Fifty-two respondents provided substantive open-ended responses and 21 semi-structured interviews were conducted. Four themes were developed (Table 5).

**Table 5:** Themes developed from open-ended survey responses (N=52) and semi-structured interviews (N=21) with undergraduates, at one university in Hong Kong Special Administrative Region, China, 2026

*Theme*	*Summary of finding*	*Illustrative pattern*
**1. Initiation and reasons for use or non-use**	Curiosity and peer offers were the pathways to initiation most often described by users; participants who did not use either product described health aversion, family values and sensory dislike.	Users described friends offering or purchasing e-cigarettes on their behalf, whereas non-users emphasized smell, health concerns and lack of need.
**2. Perceptions of harm and product comparison**	Conventional cigarettes were described as delivering a stronger nicotine effect; e-cigarettes were described as discreet and flavored, but often as more harmful because of unknown chemical contents.	Participants described e-cigarettes as easier to hide and more varied in taste, but also as potentially riskier because ingredients were uncertain.
**3. Psychosocial and social influences**	Peer norms and stress recurred as the central accounts of use; participants who did not use e-cigarettes described deliberate avoidance and mild stigma.	Respondents linked vaping to friendship circles, social situations and pressure, and some described using e-cigarettes to manage negative emotions.
**4. Views on regulation, purchasing channels and suggestions**	Views on regulation were divided: participants who did not use e-cigarettes generally supported enforcement, whereas users described the ban as displacing supply to informal markets or towards conventional cigarettes.	Reported purchasing channels included friends, online searches, WeChat contacts and crossborder sourcing from Mainland China after the 2022 ban.

Fifty-two of 267 survey respondents (19.5%) provided at least one substantive open-ended response.

*Theme 1: Initiation and reasons for use or non-use*

Participants who had used e-cigarettes described initiation as driven by curiosity and social exposure, most often through a friend who offered a device or purchased one on their behalf. Continued use was linked to academic and personal pressure; one dual user described turning to e-cigarettes under stress while wishing to avoid conventional cigarettes. Participants who did not use either product cited aversion to the smell, health concerns, family values and an absence of any perceived need.

*Theme 2: Perceptions of harm and product comparison*

Conventional cigarettes were described as delivering a stronger nicotine effect, while e-cigarettes were described as more discreet, more varied in flavor and easier to conceal. Several participants nevertheless regarded e-cigarettes as potentially more harmful because the chemical contents were unknown and because the devices were easy to use repeatedly.

*Theme 3: Psychosocial and social influences*

Peer norms and stress recurred as the central accounts of use. Participants linked vaping to friendship circles, social occasions and situational pressure, and several described using e-cigarettes to manage negative emotions. Participants who did not use e-cigarettes described deliberate avoidance of settings where vaping occurred and reported mild stigma attached to student users.

*Theme 4: Views on regulation, purchasing channels and suggestions*

Interview accounts indicated that regulation had altered purchasing and use practices rather than ending them. Participants described obtaining products through friends, WeChat contacts, online searches and cross-border sourcing from Mainland China, and described concealment strategies intended to reduce visibility in dormitories and public settings. Views on regulation were divided, and this division was also visible in the survey data: respondents who had ever used e-cigarettes were less supportive of a complete sales ban than those who had not (3.04 vs 3.96; t= -3.82, p<0.001) and more supportive of permitting adult use in designated places (4.23 vs 3.49; t=4.31, p<0.001).

### Integration of quantitative and qualitative findings

The two components converged. Peer influence, the variable most strongly associated with both outcomes in the regression models, corresponded to the accounts participants gave of peer-offer situations and social normalization, and to the survey finding that friends were the single most common acquisition channel. The absence of any difference in perceived regulatory deterrence between users and non-users, alongside the inverse association between perceived surveillance and current use, corresponded to accounts in which the restrictions were described as changing where and how products were obtained and used rather than as ending use.

## DISCUSSION

This study examined e-cigarette use among undergraduates at one university in Hong Kong, in a setting characterized by stringent regulation of alternative smoking products. Current use was lower than figures reported from less restrictive jurisdictions, where past-30-day use among high school students has reached 20.8%^2^, but higher than earlier Hong Kong estimates for secondary school students, among whom past-30-day use was 1.1%^9^, and broadly comparable with the 4.8% current use and 16.1% lifetime ever use reported for Hong Kong young adults aged 18–35 years^10^. The higher lifetime figure observed here may reflect the older age range of the present sample and the convenience sampling approach, and is consistent with the observation that experimentation persists in Hong Kong despite restrictions^10,11^.

Peer influence was the factor most strongly associated with both outcomes. This is consistent with systematic reviews of TPB applications to e-cigarette use, which find subjective and descriptive norms to be among the constructs most consistently associated with intention and use among adolescents^14^, with meta-analytic evidence for the model in smoking research more generally^15^, with the finding that adolescents who perceive e-cigarette use as common or acceptable among peers report higher use and intention^17,18^, and with socio-ecological reviews identifying peer factors as recurrent^19^. The finding is reinforced by the descriptive data, in which friends were the source of e-cigarettes for more than two-thirds of ever users and peer influence was among the two most commonly endorsed reasons for use.

Acceptability of use in public settings was associated with current use but not with retrospectively reported use during adolescence. This is consistent with the TPB proposition that attitudes towards a behavior are associated with its performance^13,14^, and with evidence that adolescents perceive e-cigarette use as more acceptable in indoor and outdoor settings than conventional smoking^18^. The difference between the two outcomes may reflect the fact that current attitudes were measured concurrently with current use but several years after the adolescent behavior, and should not be read as evidence that attitudes are relevant only to continued use.

Female sex was inversely associated with use during ages 13–19 years but not with current use. The adolescent finding is consistent with the male predominance reported among Hong Kong secondary school students^9^, while the absence of a gender difference in current use is compatible with a narrowing of the gender gap in this older sample, although the small number of events means this comparison is imprecise.

Regarding the two regulatory constructs, perceived regulatory deterrence did not differ between users and non-users, whereas perceived surveillance showed a modest inverse association with current use. One interpretation, consistent with the qualitative accounts, is that in a setting where formal retail access has already been removed, the expectation of being seen is associated with where the behavior occurs, while the expectation of punishment is not associated with whether it occurs at all. Comparative work indicates that the regulatory environment is associated with patterns and outcomes of e-cigarette use across countries^28^, and analyses of the Hong Kong youth substance-use context have described a comparable displacement of use towards less visible settings under tightening prohibition^30^. The persistence of informal supply through friends, online platforms and cross-border channels described by participants here parallels earlier Hong Kong observations that some young people obtain e-cigarettes through friends or online sources^9,10^. Discussion of the appropriate policy balance in this area remains contested^29^.

A further observation is the extent to which e-cigarette use co-occurred with conventional cigarette smoking: 86.8% of respondents who had ever used an e-cigarette had also ever smoked, and only 3.3% of those who had never used an e-cigarette had ever smoked. In this sample, therefore, e-cigarette use was very largely confined to students who also smoked, a pattern that differs from settings in which exclusive e-cigarette use among never smokers is common^2,5^. The present cross-sectional data cannot determine the order in which the two behaviors began, and the very strong association meant that models additionally adjusting for smoking were close to separation and could not usefully separate the two.

Because this study is cross-sectional, the associations reported here cannot establish temporal order or causal direction. Rather than proposing specific interventions, the findings indicate where further evidence is required. Longitudinal studies measuring peer norms, attitudes and regulatory perceptions repeatedly would be needed to establish whether these factors precede initiation and continuation among university students, and to disentangle the sequence of e-cigarette and conventional cigarette use. Multi-institution studies with probability sampling and larger numbers of users would be needed to produce prevalence estimates generalizable to the Hong Kong undergraduate population and to support models containing more variables. Studies designed specifically to characterize informal supply networks – including cross-border and messaging-platform channels – would be needed before conclusions could be drawn about their contribution to access. Evaluation studies, including controlled designs where feasible, would be required before any statement could be made about the effectiveness of peer-based or stress-focused campus programs in this setting.

### Limitations

This study has several limitations. First, convenience sampling within a single institution introduces selection bias: students with stronger views about, or greater experience of, vaping may have been more likely to respond, and the findings may not be generalizable beyond undergraduates at the participating university. Second, all measures were self-reported, which may introduce misclassification of both the exposures and the outcomes; in a legally sensitive area, such misclassification is unlikely to be non-differential, and under-reporting of use and of informal or illicit acquisition channels is plausible. Recall error is an additional concern for the retrospective item covering ages 13–19 years. Third, the cross-sectional design does not permit inference about temporal order. Fourth, several item blocks carried a ‘skip if not applicable’ instruction, which produced strongly differential non-response: perceived harm, stress-coping, stigma and deterrence items were answered by 87–94% of ever users but by only 14–48% of never users. These variables could therefore not be used as explanatory variables, and the corresponding descriptive comparisons are subject to selection. This is an important design lesson for future questionnaires in this field, in which such blocks should be presented to all respondents with a hypothetical framing. Fifth, the two perceived-harm items did not form a reliable scale (r= -0.02) and had to be reported separately, so this study provides only weak measurement of harm perceptions. Sixth, the numbers of outcome events were small (32 and 23), which limited the number of variables that could be included and produced wide confidence intervals; the models should be regarded as exploratory. Finally, although the models adjusted for age and gender, residual confounding by unmeasured factors – for example mental health status, disposable income, sensation seeking or exposure to e-cigarette marketing^26^ – cannot be excluded.

## CONCLUSIONS

E-cigarette use was reported by undergraduates at one university in Hong Kong despite a highly restrictive regulatory environment, and was very largely concentrated among students who had also smoked conventional cigarettes. Peer influence was the factor most strongly associated with both ever use during adolescence and current use, and acceptability of use in public settings was associated with current use. Perceived regulatory deterrence did not differ between users and non-users, and perceived surveillance showed only a modest inverse association with current use. Because of the cross-sectional design, the small number of users and the differential non-response affecting several measures, these associations cannot be interpreted causally. Longitudinal and multi-institution studies, together with research designed to characterize informal supply channels, are needed to establish sufficient evidence on e-cigarette use among university students in restrictive policy environments.

## Supplementary Material



## Data Availability

The data supporting this research can be found in the Supplementary file.
