# Supplementary material for: Perceptions and psychosocial correlates of e-cigarette use among Hong Kong university students: A mixed-methods study
Source: Tob Induc Dis. 2026 Oct 22;24:10.18332/tid/226575. doi: 10.18332/tid/226575 (PMC13690069; doi:10.18332/tid/226575)
Supplement: Supplementary file 1 [file TID-24-182-s1.pdf]

Tobacco Induced Diseases

Supplementary file

© 2026 Mai Z.

DOI:
10.18332/tid/226575

The content has been provided by the author(s) and has not been reviewed, verified, or endorsed by European Publishing. It may not have undergone peer review. The views, opinions, and recommendations expressed are solely those of the author(s) and do not necessarily reflect the position of European Publishing. European Publishing accepts no responsibility or liability for any consequences arising from the use of, or reliance on, this content.

Supplementary file 1. Survey questionnaire

Perception of E-cigarette Use and Its Psychosocial Factors among Hong Kong University Students: A Study of Hong Kong University Undergraduates
香港的大學本科生對電子煙使用及其心理社會因素的認知

Questionnaire Instructions 問卷說明:

Dear Respondent, Hello! This questionnaire aims to explore Hong Kong university students' perceptions of e-cigarette use and its associated psychosocial factors. Your responses will be kept completely anonymous and will be used solely for academic research purposes. No personally identifiable information will be collected or used for any commercial purposes. The questionnaire will take approximately 5–10 minutes to complete. Please answer based on your true thoughts and experiences — your input is extremely valuable to this study. Thank you very much for your time and participation in supporting academic research!

親愛的受訪者，您好！本問卷旨在了解香港本地大學生對電子煙（E-cigarette）使用的認知及其相關心理社會因素。您的回答將完全匿名處理，僅用於學術研究目的，不會作任何個人識別或商業用途。完成本問卷大約需時 5－10 分鐘。請根據您的真實想法及經驗作答，您的意見對本研究非常寶貴。感謝您撥冗參與，支持學術研究！

A. Background and Demographic Information 背景及人口統計資料

1. 1. What is your gender? 您的性別是？

Select one

◯ Male (男)

◯ Female (女)

◯ Prefer not to say (不願透露)

◯ Other: ____________________________________

2. 2. What is your age? (in years) 您的年齡是？（歲）

____

3. 3. Which major are you currently enrolled in? 您目前就讀的主修是？

Select one

◯ Chinese Language and Literature 中國語言文學

◯ English 英國語言文學

◯ History 歷史學

◯ Journalism and Mass Communication 新聞與傳播

◯ Media Design and Immersive Technology 媒體設計與虛擬實境科技

◯ Business Administration (Digital Marketing, Human Resources Management – Applied Psychology) 工商管理學 (數碼市場學、人力資源管理與應用心理學)

◯ Economics and Finance 經濟及金融

◯ Law and Business 法律與商業

◯ Accounting 會計學

◯ Financial Technology 金融科技

◯ Counselling and Psychology 輔導及心理學

◯ Psychology 心理學
◯ Sociology 社會學
◯ Arts, Culture and Technology 藝術、文化及科技
◯ Social Work 社會工作
◯ Forensic and Investigative Psychology 法證及偵緝心理學
◯ Digital Solutions for Entertainment and Game Applications 娛樂及遊戲應用數碼方案
◯ Applied Data Science 應用數據科學

4. 4. Which year of study are you currently in? 您目前就讀的年級是？
Select one

◯ Year 1 (一年級)
◯ Year 2 (二年級)
◯ Year 3 (三年級)
◯ Year 4 (四年級)

5. 5. Where did you mainly attend secondary school (Form 1–6 or equivalent)? 您主要在中學階段（中一至中六或同等）就讀的地點是？
Select one

◯ Hong Kong SAR (香港)
◯ Mainland China (中國內地)
◯ Other: ____

6. 6. Who do you mainly live with at present? 您目前主要與誰同住？
Select one

◯ Family (家人)
◯ Student hostel / shared flat (學生宿舍 / 合租公寓)
◯ Alone (獨居)
◯ Other: ____

B. Smoking and E-Cigarette Use Behaviors 吸煙及電子煙使用行為

B1. Conventional Cigarette Use 傳統香煙使用

7. 1. Have you ever smoked a conventional cigarette (even just once or twice)? 您是否曾經吸過傳統香煙（即使只是一兩次）？
Select one

◯ Yes (是)
◯ No (否)

8. 2. During the past 30 days, on how many days did you smoke conventional cigarettes? (0–30 days) 在過去 30 天內，您吸傳統香煙的天數是多少？（0–30 天）

____

B2. E-Cigarette Use During Teenage Years 青少年時期電子煙使用

9. 3. Between the ages of 13 and 19 (secondary school / pre-university), did you ever use an e-cigarette (even just once or twice)? 在 13 至 19 歲期間（中學 / 大學前），您是否曾經使用電子煙（即使只是一兩次）？

Select one

◯ Yes (是)

◯ No (否)

◯ Cannot remember (不記得)

10. 4. (If Yes to Q3) During the ages of 13–19, how often did you usually use e-cigarettes?（若 Q3 回答是）在 13 至 19 歲期間，您通常使用電子煙的頻率是？

Select one

◯ Only once or a few times (只一次或幾次)

◯ Less than once a month (少於每月一次)

◯ 1–3 times per month (每月 1–3 次)

◯ 1–6 times per week (每週 1–6 次)

◯ Almost every day (幾乎每天)

11. 5. How old were you when you first tried an e-cigarette? 您第一次嘗試電子煙時的年齡是？

____

B3. Current E-Cigarette Use (University Period) 目前電子煙使用（大學時期）

12. 6. During the past 30 days, on how many days did you use an e-cigarette? (0–30 days)在過去 30 天內，您使用電子煙的天數是多少？（0–30 天）

____

13. 7. In what situations or places do you mainly use e-cigarettes? (You may select more than one) 您主要在哪些情況或地點使用電子煙？（可選多項）

Select all that apply

▢ At home (在家)

▢ At a friend's home (朋友家)

▢ Near or around campus (校園附近)

▢ Bars / restaurants (酒吧 / 餐廳)

▢ Public places (e.g., streets, parks) (公共場所，例如街道、公園)

▢ Other: ____________________________________

14. 8. What are your main reasons for using e-cigarettes? (You may select more than one) 您使用電子煙的主要原因是？（可選多項）

Select all that apply

- ▢ Curiosity (好奇)
- ▢ Fun / fashion / trend (有趣 / 時尚 / 潮流)
- ▢ Stress relief / relaxation (減壓 / 放鬆)
- ▢ To help quit conventional cigarettes (幫助戒傳統香煙)
- ▢ Like the taste / enjoy the variety of flavors (喜歡味道 / 享受多樣口味)
- ▢ Friends use them (peer influence) (朋友使用（同儕影響）)
- ▢ Believe they are less harmful than conventional cigarettes (相信比傳統香煙危害較小)
- ▢ Cheaper / easier to access (更便宜 / 更容易取得)
- ▢ Other: ______________________________________

15. 9. How do you mainly obtain e-cigarettes? (You may select more than one) 您主要如何取得電子煙？（可選多項）

Select all that apply

- ▢ Friends gave / friends bought for me (朋友給予 / 朋友代購)
- ▢ Online platforms (e.g., social media, online shops) (網上平台，例如社交媒體、網店)
- ▢ Physical shops (before the ban) (實體店（禁令前）)
- ▢ Parallel imports / black market channels (平行進口 / 黑市渠道)
- ▢ Other: ______________________________________

D. Attitudes and Perceptions toward E-Cigarette Use 對電子煙使用的態度及認知

D1. Acceptance of Use in Different Public Settings 在不同公共場所使用的接受度

1. To what extent do you accept the use of e-cigarettes in the following places? (1 = Totally unacceptable to 5 = Totally acceptable)
您對在以下場所使用電子煙的接受程度是？（1 = 完全不可接受 至 5 = 完全可接受）

16. On the street / in parks (街道 / 公園)

◯ 1 ◯ 2 ◯ 3 ◯ 4 ◯ 5

17. In restaurants / cafés (餐廳 / 咖啡廳)

◯ 1 ◯ 2 ◯ 3 ◯ 4 ◯ 5

18. In outdoor areas on campus (校園戶外區域)

◯ 1 ◯ 2 ◯ 3 ◯ 4 ◯ 5

19. In indoor public places (e.g., shopping malls, near MTR station entrances) (室內公共場所，例如購物中心、地鐵站入口附近)

◯ 1 ◯ 2 ◯ 3 ◯ 4 ◯ 5

2. Please indicate your level of agreement (1 = Strongly disagree to 5 = Strongly agree):
請指明您的同意程度（1 = 強烈不同意 至 5 = 強烈同意）：

20. As long as it does not disturb others, using e-cigarettes in areas where smoking is banned is acceptable. (只要不打擾他人，在禁煙區使用電子煙是可接受的。)

◯ 1 ◯ 2 ◯ 3 ◯ 4 ◯ 5

21. It is acceptable to use e-cigarettes in places where there are children or pregnant women. (在有兒童或孕婦的地方使用電子煙是可接受的。)

◯ 1 ◯ 2 ◯ 3 ◯ 4 ◯ 5

D2. Perceived Physical and Psychological Impact (Those who are not applicable, please skip) 感知的身體及心理影響 (不適用者請跳過)

3. Please indicate your level of agreement (1 = Strongly disagree to 5 = Strongly agree):
請指明您的同意程度（1 = 強烈不同意 至 5 = 強烈同意）：

22. Using e-cigarettes helps me relax. (使用電子煙幫助我放鬆。)

◯ 1 ◯ 2 ◯ 3 ◯ 4 ◯ 5

23. I worry about my health if I use e-cigarettes for a long time. (如果長期使用電子煙，我會擔心健康。)

◯ 1 ◯ 2 ◯ 3 ◯ 4 ◯ 5

24. Using e-cigarettes helps me concentrate or study. (使用電子煙幫助我集中精神或學習。)

◯ 1 ◯ 2 ◯ 3 ◯ 4 ◯ 5

25. I feel that e-cigarettes have little impact on my physical and mental health. (我覺得電子煙對我的身心健康影響不大。)

◯ 1 ◯ 2 ◯ 3 ◯ 4 ◯ 5

E. Peers, Family, and Stigma / Concealment 同儕、家庭及污名 / 隱瞞

1. Please indicate your level of agreement (1 = Strongly disagree to 5 = Strongly agree):
請指明您的同意程度（1 = 強烈不同意 至 5 = 強烈同意）：

26. Many of my friends use e-cigarettes. (我的許多朋友使用電子煙。)

◯ 1 ◯ 2 ◯ 3 ◯ 4 ◯ 5

27. The more friends around me who use e-cigarettes, the more I want to try or continue using them. (周圍朋友使用電子煙越多，我越想嘗試或繼續使用。)

◯ 1 ◯ 2 ◯ 3 ◯ 4 ◯ 5

28. If my friends invite me to vape, it is hard for me to say no. (如果朋友邀請我吸電子煙，我很難拒絕。)

◯ 1 ◯ 2 ◯ 3 ◯ 4 ◯ 5

29. 2. Does anyone in your family regularly smoke conventional cigarettes? 您的家人中是否有人經常吸傳統香煙？

Select one

◯ Father (父親)

◯ Mother (母親)

◯ Siblings (兄弟姊妹)

◯ None (沒有)

◯ Other: ____

30. 3. Do you have any family members or partners who use e-cigarettes? 您是否有家人或伴侶使用電子煙？

Select one

◯ Yes (是)

◯ No (否)

4. If you use or have used e-cigarettes, would you let the following people know? (If you have never used e-cigarettes, please choose 'Not applicable'.)
如果您使用或曾使用電子煙，您會讓以下人士知道嗎？（若從未使用，請選擇「不適用」。）

31. Parents / family members (父母 / 家人)

Select one

◯ Definitely would tell (一定會告訴)

◯ Depends on the situation (視情況而定)

◯ Would not tell (不會告訴)

◯ Not applicable (不適用)

32. Close friends (親密朋友)

Select one

◯ Definitely would tell (一定會告訴)

◯ Depends on the situation (視情況而定)

◯ Would not tell (不會告訴)

◯ Not applicable (不適用)

33. Teachers / university staff (老師 / 大學職員)

Select one

◯ Definitely would tell (一定會告訴)

◯ Depends on the situation (視情況而定)

◯ Would not tell (不會告訴)

◯ Not applicable (不適用)

34. Partner (伴侶)

Select one

◯ Definitely would tell (一定會告訴)

◯ Depends on the situation (視情況而定)

◯ Would not tell (不會告訴)

◯ Not applicable (不適用)

5. Please indicate your level of agreement (1 = Strongly disagree to 5 = Strongly agree) (Those who are not applicable, please skip)
請指明您的同意程度（1 = 強烈不同意 至 5 = 強烈同意）（不適用者請跳過）

35. If my family found out that I use e-cigarettes, they would see me as a 'bad boy/girl'. (如果家人發現我使用電子煙，他們會視我為「壞男孩/女孩」。

◯ 1 ◯ 2 ◯ 3 ◯ 4 ◯ 5

36. I worry that if teachers or the university find out I use e-cigarettes, they will label me negatively. (我擔心如果老師或大學發現我使用電子煙，他們會給我負面標籤。)

◯ 1 ◯ 2 ◯ 3 ◯ 4 ◯ 5

37. Society generally views university students who use e-cigarettes as less self-disciplined. (社會一般視使用電子煙的大學生為較缺乏自律。)

◯ 1 ◯ 2 ◯ 3 ◯ 4 ◯ 5

38. In the past month, I have often felt very stressed. (在過去一個月，我經常感到很大壓力。)

◯ 1 ◯ 2 ◯ 3 ◯ 4 ◯ 5

39. I sometimes use e-cigarettes to cope with negative emotions. (我有時使用電子煙來應對負面情緒。)

◯ 1 ◯ 2 ◯ 3 ◯ 4 ◯ 5

F. Policy Awareness, Deterrence, and Surveillance 政策認知、阻嚇及監察

40. 1. Are you aware that Hong Kong has banned the import, sale, and promotion of e-cigarettes? 您是否知道香港已禁止進口、銷售及推廣電子煙？

Select one

◯ Yes (是)

◯ No (否)

◯ Have heard of it but not sure of the details (聽過但不確定細節)

2. Please indicate your level of agreement (1 = Strongly disagree to 5 = Strongly agree) (Those who are not applicable, please skip)
請指明您的同意程度（1 = 強烈不同意 至 5 = 強烈同意）（不適用者請跳過）

41. Because I am worried about being fined or prosecuted, I have reduced or stopped using e-cigarettes. (因為擔心被罰款或起訴，我已減少或停止使用電子煙。)

◯ 1 ◯ 2 ◯ 3 ◯ 4 ◯ 5

42. The government's tougher stance on e-cigarettes makes me less willing to use them in public. (政府對電子煙的更嚴厲立場使我不願在公共場所使用它們。)

◯ 1 ◯ 2 ◯ 3 ◯ 4 ◯ 5

43. If I use e-cigarettes in a no-smoking area, I think there is a high chance of being noticed or reported. (如果我在禁煙區使用電子煙，我認為被發現或舉報的機會很高。)

◯ 1 ◯ 2 ◯ 3 ◯ 4 ◯ 5

44. I feel that the university/government closely monitors the use of e-cigarettes in public places. (我覺得大學/政府密切監察公共場所的電子煙使用。)

◯ 1 ◯ 2 ◯ 3 ◯ 4 ◯ 5

45. I support a complete ban on the sale of e-cigarettes in Hong Kong. (我支持在香港完全禁止銷售電子煙。)

◯ 1 ◯ 2 ◯ 3 ◯ 4 ◯ 5

46. Adults should be allowed to use e-cigarettes legally in designated places. (成人應允許在指定場所合法使用電子煙。)

◯ 1 ◯ 2 ◯ 3 ◯ 4 ◯ 5

47. The government should focus more on education rather than relying mainly on strict penalties. (政府應更多注重教育，而不是主要依賴嚴格罰則。)

◯ 1 ◯ 2 ◯ 3 ◯ 4 ◯ 5

G. Open-Ended Questions and Qualitative Data 開放式問題及質性資料

48. 1. Please briefly describe your reasons for starting (or having started) to use e-cigarettes and how this has affected your life.
請簡述您開始（或曾開始）使用電子煙的原因，以及這如何影響您的生活。

____

____

49. 2. What are your views on the current and upcoming e-cigarette regulations in Hong Kong? Do you have any suggestions?
您對香港目前及即將實施的電子煙規管有何看法？您有任何建議嗎？

____

____

50. Follow-Up Interview Invitation 後續訪談邀請

If you are willing to participate in a follow-up semi-structured interview to discuss your experiences in more depth, please leave your contact information (e.g., email or phone number). The interview will last 15-30 minutes and will be conducted at a convenient time. As a token of appreciation, you will receive a small cash reward. Your contact details will be kept confidential and used only for scheduling the interview.
如果您願意參與後續半結構化訪談，以更深入討論您的經驗，請留下您的聯絡方式（例如電郵或電話號碼）。訪談將持續 15-30 分鐘，並在方便時間進行。作為感謝，您將獲得少許現金酬謝。您的聯絡資料將保密，僅用於安排訪談。

Select one

◯ Yes, I am willing. (是，我願意。)

◯ No, thank you. (否，謝謝。)

51. Following the previous question, choose "Yes, I am willing" Contact: (e.g., email or phone number)
接上一條問題，選擇「是，我願意。」聯絡方式：（例如電郵或電話號碼）

____

Supplementary file 2. Semi-structured interview guide

Semi-structured interview guide for follow-up interviews with undergraduate students

Purpose of the interview

This interview guide was developed for the qualitative phase of a sequential explanatory mixed-methods study examining perceptions of e-cigarette use and its psychosocial correlates among undergraduate students in Hong Kong. The guide was designed to explore participants' views on e-cigarette use, social and psychological influences, perceived harms, and awareness of Hong Kong's alternative smoking product regulations.

Target participants

Participants were full-time undergraduate students aged 18 years or above who had completed the survey and agreed to be contacted for a follow-up interview.

Estimated duration

Approximately 15–30 minutes.

Instructions for interviewer

The interviewer should use the questions flexibly and follow the participant's responses where appropriate. Probes may be used to clarify meaning, elicit examples, and explore unexpected issues in greater depth. Questions should be asked in a neutral, non-judgmental manner. Participants may decline to answer any question or stop the interview at any time.

Opening script

Thank you for agreeing to take part in this interview. The purpose of this interview is to better understand university students' views and experiences related to e-cigarettes, including social influences, perceptions of harm, and views on current regulations. Your participation is voluntary. The interview will be kept confidential, and your name will not appear in any report or publication. With your permission, the interview will be audio-recorded for transcription and analysis. You may skip any question or stop the interview at any time.

Before we begin:

1. Have you read the participant information and consented to take part in this interview?
2. Do you agree to audio recording?
3. Do you have any questions before we start?

Section 1. Background and general context

1. Could you briefly tell me about yourself, such as your year of study and living arrangement?

Possible probes: local or non-local background; living with family, in hostel, or shared accommodation.

2. In your view, how common is e-cigarette use among university students in Hong Kong?

Possible probes: among friends, classmates, hostel residents, or people of similar age.

3. What kinds of attitudes toward e-cigarettes do you usually see among students around you?

Possible probes: acceptable/unacceptable, fashionable/unfashionable, normal/stigmatized.

Section 2. Personal experience and use or non-use

4. Have you ever used e-cigarettes, even once, or been in situations where people around you were using them?

Possible probes: first exposure, first trial, social settings, school period versus university period.

5. If yes, can you describe how you first came into contact with e-cigarettes?

Possible probes: curiosity, friends, social activities, stress, convenience, flavours.

6. If no, what are the main reasons you have not used e-cigarettes?

Possible probes: health concerns, family influence, dislike of smell, legal concerns, lack of interest.

7. What do you think usually motivates students to try or continue using e-cigarettes?

Possible probes: curiosity, stress relief, emotional coping, peer influence, fun, taste, concealability.

Section 3. Perceptions of harm and product comparison

8. How harmful do you think e-cigarettes are compared with conventional cigarettes?

Possible probes: less harmful, equally harmful, more harmful; short-term versus long-term effects.

9. What comes to mind when you think about the ingredients or chemicals in e-cigarettes?

Possible probes: nicotine, flavourings, uncertainty about contents, addiction risk.

10. How addictive do you think e-cigarettes are?

Possible probes: compared with cigarettes; easy or difficult to stop; signs of dependence.

11. In your opinion, what are the main differences between e-cigarettes and conventional cigarettes?

Possible probes: smell, convenience, concealment, health risk, price, social image.

Section 4. Social and psychosocial influences

12. How much do friends or peers influence e-cigarette use among students?

Possible probes: being offered by friends, social pressure, wanting to fit in, seeing others use them.

13. How much does family influence students' decisions about e-cigarette use?

Possible probes: family smoking or vaping, parental disapproval, family values.

14. Do you think stress, academic pressure, or negative emotions play a role in e-cigarette use?

Possible probes: coping with stress, relaxing, concentrating, boredom, loneliness.

15. How easy or difficult do you think it is for students to refuse e-cigarettes if offered by friends?

Possible probes: confidence in saying no, situational pressure, repeated exposure.

Section 5. Access, regulation, and enforcement

16. How aware are you of Hong Kong's regulations on e-cigarettes and other alternative smoking products?

Possible probes: ban on import, sale, promotion, manufacture, and public possession.

17. Do these regulations affect whether students use or carry e-cigarettes? Why or why not?

Possible probes: fear of penalties, inconvenience, secrecy, shift to hidden use.

18. In your understanding, how do students currently obtain e-cigarettes despite the ban?

Possible probes: friends, online channels, social media, cross-border sourcing, informal networks.

19. Do you think students feel monitored or worried about being caught when using or carrying e-cigarettes?

Possible probes: on campus, in public places, near transport stations, in hostels.

20. In your view, has the ban reduced use, or has it mainly changed how people get and use e-cigarettes?

Possible probes: black market, concealment, substitution, reduced visibility.

Section 6. Campus and policy suggestions

21. What do you think universities should do to reduce e-cigarette use among students?

Possible probes: education, counselling, peer-led programmes, stress-management support, clearer rules.

22. What do you think the government should do regarding e-cigarettes?

Possible probes: stricter enforcement, education campaigns, harm-reduction approach, youth-focused prevention.

23. Do you think current policies are fair and effective? Why or why not?

Possible probes: effectiveness, unintended consequences, practicality, student perspectives.

Closing question

24. Is there anything else you would like to add about e-cigarette use, student life, or the current policy environment that we have not discussed?

Supplementary file 3.

Qualitative Codebook / Coding Framework

Study: Perceptions and psychosocial factors associated with e-cigarette use among university students in Hong Kong: A mixed-methods study

Data sources: Semi-structured interview transcripts (verbatim, de-identified) and open-ended survey responses.

Analytic approach: Inductive thematic analysis. Transcripts were read line by line and initial (open) codes were assigned to meaningful units of text. Related open codes were grouped into sub-themes, which were further organised into four overarching themes. Coding was conducted by the author, with peer debriefing with the research supervisor used to strengthen trustworthiness.

Note: this document is a coding framework for methodological transparency. It does not reproduce verbatim transcript text; illustrative descriptions are paraphrased and stripped of details that could identify individual participants (e.g., specific countries/cities of exchange study, specific family members, specific dates/prices), consistent with the interview consent terms.

Theme 1 — Initiation and reasons for use or non-use

Open code	Definition	Illustrative pattern (paraphrased)
Peer offer as entry point	First use occurred because a friend or classmate directly handed over or offered a cigarette/e-cigarette	Several participants described a friend or senior classmate simply passing them a device or cigarette in a social setting, without having sought it out themselves
Curiosity / novelty	Motivation framed as wanting to know what the experience was like, rather than a specific need	Participants described trying the product “just to see,” often prompted by novelty (e.g., fruit flavours, unfamiliar device)
Stress/emotional trigger	First or continued use linked to a specific stressful or emotionally difficult event (exam failure, breakup, academic pressure)	One participant described being offered a cigarette by a friend immediately after a poor exam result and crying in a washroom; another linked first cigarette use to a breakup

Open code	Definition	Illustrative pattern (paraphrased)
Overseas/exchange exposure	First exposure occurred while studying or travelling abroad, where e-cigarette use was more normalised or accessible	A participant described first trying e-cigarettes during an overseas exchange programme, where use among peers was common and open
Early/pre-university access	First contact with e-cigarettes occurred in childhood or early adolescence via informal retail access (e.g., a neighbourhood shop) before formal restriction	One participant described purchasing an e-cigarette from a local convenience shop in late primary school, before the product was tightly regulated
Family exposure without initiation	Participant grew up with a smoking family member but did not take up use themselves	Multiple non-users described a father or grandfather who smoked, with the participant reporting discomfort rather than uptake
Health-based abstention	Explicit reasoning that use was avoided because of concern about respiratory or other health effects, sometimes tied to a personal or family medical history	Some participants cited a personal history of respiratory illness or family illness as their main reason for avoiding both products
Family values / upbringing	Non-use attributed to explicit family teaching or a conservative household norm against smoking	Several non-users described their families as having "never allowed" smoking and having instilled this as a value from a young age
Sensory aversion	Dislike of the smell or physical sensation of smoke as a stated reason for non-use or for avoiding smokers	A recurring reason for non-use was strong personal dislike of the smell of smoke, described as being present "since childhood"
No perceived need/motivation	Non-use explained by an absence of any functional reason to use, rather than active moral opposition	One non-user stated they simply saw no purpose the product could serve for them and considered it an unnecessary expense
Cost as deterrent	Price of cigarettes or e-cigarettes cited as a practical reason to avoid or limit use	A few participants raised the cost of tobacco products, including Hong Kong's tobacco duty, as a factor in their own or others' decisions

Theme 2 — Perceptions of harm and product comparison

Open code	Definition	Illustrative pattern (paraphrased)
E-cigarette as "milder" sensory experience	E-cigarettes described as less harsh, less irritating, or more pleasant-tasting than cigarettes	Several ever-users described e-cigarettes as "less harsh on the throat" and offering a wider range of flavours than cigarettes
Weaker nicotine "hit" from e-cigarettes	E-cigarettes seen as providing less of a nicotine effect than conventional cigarettes, sometimes cited as a reason for continuing to also smoke cigarettes	Some dual users described e-cigarettes as not giving them the same "hit" as a cigarette, especially after a long smoking history
Discreetness / concealability	E-cigarettes valued for being easier to use without detection (less smell, no visible smoke)	Participants noted that e-cigarettes leave less residual smell and can be used more discreetly indoors or in dormitories
Unknown chemical content as risk	Uncertainty about e-cigarette ingredients cited as a reason to view them as potentially equally or more harmful than cigarettes	Several participants, including some users, expressed concern that they did not know what chemicals were in e-cigarette liquid
Perceived equivalence to illicit drugs	E-cigarettes, particularly adulterated products, discussed as functionally similar to controlled drugs	One participant explicitly compared certain e-cigarette additives to a controlled substance, framing regulatory treatment of e-cigarettes as analogous to drug control
Moral relativism about "legal" harmful products	Participants argued that conventional cigarettes are similarly harmful/addictive but tolerated because of economic or political factors	One participant argued that cigarettes could equally be considered a "drug" but are not banned outright because of tax revenue and the size of the existing smoking population
Secondhand smoke as a harm to others	Concern about harming third parties, distinct from harm to the user themselves	Several non-users framed their opposition to smoking partly in terms of secondhand smoke infringing on others' choices, not just personal health risk

Theme 3 — Psychosocial and social influences

Open code	Definition	Illustrative pattern (paraphrased)
Social bonding function	Use framed as something done together with friends, reinforcing group belonging	Participants described smoking or vaping together as a shared social activity at gatherings, birthdays, or nights out
Stress-coping function	Use explicitly described as a way to manage stress or negative emotion	Multiple participants described using cigarettes or e-cigarettes to “calm down” or cope after a stressful event, particularly around exams
Non-user avoidance strategy	Non-users describing deliberate social distancing from users, or avoiding close friendships with users	Several non-users said they would not become close friends with someone who smokes or vapes, even if willing to interact normally otherwise
Mild stigma toward users	Users described as facing subtle social judgement, without being treated as outright deviant	One non-user described attaching a mild “not a good student” label to users while also acknowledging this was a personal, not universal, view
Empathy/understanding toward users despite disapproval	Non-users describing sympathy for users’ circumstances (e.g., family stress) despite personal disapproval of the behaviour	One participant described a shift from avoiding smoking classmates to understanding their stress-related reasons for smoking, without adopting the behaviour themselves
Peer network normalisation over time	Increased proportion of smoking/vaping friends after entering university compared with secondary school	Some participants noted that more of their friends smoke now than in secondary school, describing this as a “normal” shift after entering university
Loss of contact with initiating peer	The friend who introduced the participant to the product is often no longer in regular contact	Several participants noted they were no longer in touch with the friend who first offered them a cigarette or e-cigarette

Theme 4 — Views on regulation, purchasing channels, and suggestions

Open code	Definition	Illustrative pattern (paraphrased)
Low perceived enforcement risk	Belief that the practical chance of being caught or penalised is low, despite awareness of the law	Participants described the ban as something they were aware of but did not expect to be actively enforced against them personally
Ban perceived as displacing rather than eliminating use	View that prohibition pushes use towards informal or black-market channels rather than stopping it	Several participants argued that banning e-cigarettes outright increases black-market activity and informal peer-to-peer supply rather than reducing use
Cross-border/informal acquisition	Description of obtaining e-cigarettes via Mainland China, online platforms, or messaging-app contacts	Participants described purchasing e-cigarettes through online platforms or bringing them across the border, sometimes noting a preference for products with formal licensing
Tax/price substitution argument	Argument that high cigarette taxation pushes users toward (cheaper or comparably priced) e-cigarettes	Some participants argued that Hong Kong's high tobacco duty makes e-cigarettes a comparably priced, and therefore attractive, substitute
Support for graduated/education-first approach	Preference for public education and gradual behaviour change over strict punitive enforcement	Several participants suggested that sustained public education about e-cigarette risks should precede or accompany stricter enforcement
Support for stricter enforcement/penalties	Preference for more active policing, fines, or reporting mechanisms	Other participants suggested concrete enforcement mechanisms, such as designated patrols or public reporting channels with fines
Perceived double standard (jurisdictional)	Observation that e-cigarettes are legal or tolerated elsewhere (e.g., across the border) while banned in Hong Kong	Several participants contrasted Hong Kong's strict ban with more permissive regulation elsewhere, questioning the consistency of the policy
Campus-level policy	Awareness of university-	A few participants referenced

Open code	Definition	Illustrative pattern (paraphrased)
awareness	specific signage or enforcement regarding smoking/vaping on campus	having seen campus notices about fines for e-cigarette use on university grounds

Summary table: Theme-to-code mapping (for Table 5 of the manuscript)

Manuscript theme	Number of distinct open codes identified
Theme 1: Initiation and reasons for use or non-use	11
Theme 2: Perceptions of harm and product comparison	7
Theme 3: Psychosocial and social influences	7
Theme 4: Views on regulation, purchasing channels, and suggestions	8

Note on interview subsample composition

Based on coding of the 21 labelled interview transcripts, product-use status at the time of interview was distributed as follows:

Status	n
Non-user of both e-cigarettes and conventional cigarettes	10
Exclusive conventional cigarette user	1
Dual user (ever used both products)	10
Exclusive e-cigarette user	0
